# Development of 15 nuclear EST microsatellite markers for the paleoendemic conifer *Pherosphaera hookeriana* (Podocarpaceae)

**DOI:** 10.1002/aps3.1160

**Published:** 2018-06-26

**Authors:** James R. P. Worth, James R. Marthick, Maurizio Rossetto, Joel Cohen, Greg Bourke, Gregory J. Jordan

**Affiliations:** ^1^ Department of Forest Molecular Genetics and Biotechnology Forestry and Forest Products Research Institute Matsunosato 1 Tsukuba Ibaraki 305‐8687 Japan; ^2^ Menzies Institute for Medical Research University of Tasmania Hobart Tasmania Australia; ^3^ National Herbarium of New South Wales Royal Botanic Gardens and Domain Trust Mrs. Macquaries Road Sydney New South Wales 2000 Australia; ^4^ Blue Mountains Botanic Garden 1–17 Tomah Drive Mount Tomah New South Wales 2758 Australia; ^5^ School of Biological Sciences University of Tasmania Private Bag 55 Hobart Tasmania Australia

**Keywords:** conifer, next‐generation sequencing, paleoendemic, *Pherosphaera*, Podocarpaceae, RNA‐seq, Tasmania

## Abstract

**Premise of the Study:**

Nuclear microsatellite markers were developed for population genetic analysis of the threatened paleoendemic conifer *Pherosphaera hookeriana* (Podocarpaceae).

**Methods and Results:**

Fifteen variable loci were identified showing one to 13 alleles per population, with seven loci displaying at least four alleles in all populations, and the average number of alleles per locus ranging from 4.80 to 5.93 per population. Levels of observed heterozygosity per locus varied from 0.00 to 0.91, while average heterozygosity across all loci varied from 0.54 to 0.63 between populations. All loci also amplified in the endangered congener *P. fitzgeraldii*, but only five of the loci had more than one allele.

**Conclusions:**

These 15 loci are the first microsatellite markers developed in the genus *Pherosphaera*. These loci will be useful for investigating the species' extant genetic diversity and structure, the impact of past environmental change, and the significance of asexual reproduction.

*Pherosphaera hookeriana* W. Archer (Mount Mawson or drooping pine) is a small, scale‐leaved conifer with a restricted range in montane areas of the high rainfall southwest region of Tasmania. The species is of particular conservation importance because of its paleoendemic status, with the genus *Pherosphaera* W. Archer estimated to have diverged 115 mya (Biffin et al., 2011) and fossils from the Eocene or Early Oligocene age in Tasmania being almost identical in morphology to extant *P. hookeriana* (Brodribb and Hill, 2004). Pollen evidence suggests that the species was an important component of the Last Glacial vegetation, having a wider distribution than present and occurring at lower elevations down to near sea level (Colhoun, 1985). At the onset of postglacial warming, the species retreated to higher altitudes or, in some cases, became locally extinct (Macphail et al., 2014). The species is classified as Vulnerable under the Tasmanian Government Threatened Species Protection Act 1995 due to its limited range and its high sensitivity to fire and drought, which have intensified in recent decades (Threatened Species Section, 2016). It is perhaps the narrow distribution of the species that has so far limited the impact of post–European‐arrival fires that have so devastated other more widespread fire‐sensitive Tasmanian endemic conifers (Marris, 2016).

*Pherosphaera hookeriana* is thought to predominantly reproduce asexually by root suckering, partly because sexual reproduction is seldom observed. However, the importance of clonality is untested. The only other member of the genus, *P. fitzgeraldii* (F. Muell.) Hook. f., is confined to sandstone ledges of waterfalls in the Blue Mountains west of Sydney, New South Wales—some 990 km north of any population of *P. hookeriana*. *Pherosphaera fitzgeraldii* is Critically Endangered according to the International Union for Conservation of Nature and Natural Resources (IUCN; Thomas, 2013), with only nine populations known. It is thought to consist of only 755 individuals (Fourt‐Wells, 2014), although the actual number of genotypes may be far lower if the dominant mode of reproduction is asexual (Jones and Llewellyn, 1993).

This study aims to develop nuclear microsatellite markers for *P. hookeriana* and to test their utility in the endangered *P. fitzgeraldii*. These markers will be useful for understanding the genetic diversity and structure of both species in this important basal Podocarpaceae genus, for examining how the current retraction to interglacial refugia of *P. hookeriana* has impacted genetic diversity, and in determining the level of clonal reproduction.

## METHODS AND RESULTS

Total RNA was extracted from an individual of *P. hookeriana* sourced from Mt. Field National Park and grown in the conifer collection of the School of Biological Sciences, University of Tasmania, using a Plant RNA Isolation Mini Kit (Agilent Technologies, Santa Clara, California, USA). An RNA‐Seq data set was constructed by the Beijing Genomics Institute on an Illumina HiSeq 4000 platform (Illumina, San Diego, California, USA). The *P. hookeriana* RNA‐Seq data consisted of 43,176,890 paired‐end reads of 100‐bp length. De novo assembly was undertaken in CLC Genomics Workbench 8.5.1 (CLC Bio, Aarhus, Denmark), and the 33,066 resultant contigs (N50 = 1728 bp) were mined for microsatellite regions (all contigs are available from the Dryad Digital Repository: https://doi.org/10.5061/dryad.br73qg2; Worth et al., 2018). Primers were developed bordering these regions with default settings using PrimerPro (http://webdocs.cs.ualberta.ca/~yifeng/primerpro/). Microsatellites were selected if they met the following criteria: the tandem repeats were >1 bp in length, repeat units exceeded >8, and the microsatellite was located >25 bp from the beginning or end of the contig. These criteria resulted in 67 microsatellite primer pairs that were trialed for amplification in four samples. A total of 53 primer pairs successfully amplified and were subsequently tested for size heterogeneity in eight samples representative of the full distribution of the species. For all loci, the forward primer was synthesized with one of three different M13 sequences (5′‐GCCTCCCTCGCGCCA‐3′, 5′‐GCCTTGCCAGCCCGC‐3′, and 5′‐CAGGACCAGGCTACCGTG‐3′), and the reverse was tagged with a PIG‐tail (5′‐GTTTCTT‐3′; Brownstein et al., 1996). The PCR reactions were performed following the standard protocol of the QIAGEN Multiplex PCR Kit (QIAGEN, Hilden, Germany) and consisted of a 10‐μL reaction volume, containing approximately 5 ng of DNA, 5 μL of 2× Multiplex PCR Master Mix, 0.06 μM of forward primer, 0.1 μM of reverse primer, and 0.08 μM of fluorescently labeled M13 primer. The PCR thermal profile consisted of an initial denaturation at 95°C for 3 min; followed by 35 cycles of 95°C for 30 s, 60°C for 3 min, 68°C for 1 min; and a 20‐min extension at 68°C. The PCR products were separated by capillary electrophoresis on an ABI 3130 Genetic Analyzer (Life Technologies, Waltham, Massachusetts, USA) with the GeneScan 600 LIZ Size Standard (Life Technologies), and genotyping was done in GeneMarker (SoftGenetics, State College, Pennsylvania, USA). Genetic analyses were undertaken in GenAlEx 6.5 (Peakall and Smouse, 2006) and GENEPOP 4.2 (Raymond and Rousset, 1995).

A total of 15 primer pairs were found to reliably amplify, show size variability, and were readily scorable (Table 1). The genetic variability of these 15 loci was examined in 94 samples from three populations of *P. hookeriana* (Gowan Brae on the Nive River, Wombat Moor from Mt. Field National Park, and The Parthenon from Cradle Mountain–Lake St. Clair National Park [one of the most northern known populations]; see Appendix 1 for more details) and nine individuals of *P. fitzgeraldii* including samples from four of the nine known populations (Appendix 2). For *P. hookeriana*, the 15 loci displayed between one and 13 alleles per population, with seven loci displaying at least four alleles in all populations (average number of alleles per locus ranged from 4.8 to 5.93 per population). The average observed heterozygosity over all populations was 0.58 (from 0.54 to 0.63) (Table 2). No significant deviations from Hardy–Weinberg equilibrium expectations were detected for any loci except for locus Phero_3893 (*P* = 0.0179). In addition, allele frequencies appeared independent among loci except for Phero_8380 and Phero_12816 (*P* < 0.0001). The 15 loci all amplified in *P. fitzgeraldii* but displayed low variation, with only five loci with more than one allele. Two loci (Phero_20099 and Phero_6366) had four alleles (Table 2), including some that may be population specific (data not shown).

**Table 1:** Characteristics of the 15 nuclear microsatellite markers developed for *Pherosphaera hookeriana*

Locus^a^	Primer sequences (5′–3′)	Repeat motif	Allele size range (bp)	BLASTX top hit description	*E*‐value	GenBank accession no.
Phero_20099	F: GAACTATTGATTAACCACCAATACAA R: GGAACCATGATTCTGATGGG	(AT)_9_	223–285	Hypothetical protein 2_1728_01 [*Pinus radiata*]	4.00E‐25	MH017850
Phero_8380	F: GAACCCAAACACAACGTTCA R: CCCGGTCTCTACTCTGATGG	(TA)_14_	248–280	Unknown [*Picea sitchensis*]	3.50E‐166	MH017842
Phero_11789	F: TATGCCTCCTCTCGAAATGC R: TCACACCATTCTATTGGTTTTCC	(GT)_8_GAGAGAGAGT(GA)_7_	172–180	Unknown [*Picea sitchensis*]	0	MH017844
Phero_18747	F: ATCCCCATGAGCTGAAACAC R: CCCTTGGCTGTCAAAAGAAA	(CAT)_10_	264–276	Unknown [*Picea sitchensis*]	2.60E‐91	MH017849
Phero_6366	F: CTAGATGTTTCCCACCCCCT R: TACCATTCCAATAGCCCAGC	(AAG)_8_	272–284	Lipid transfer‐like protein VAS [*Helianthus annuus*]	9.20E‐16	MH017840
Phero_8339	F: CATAGCAGTTGCGAGCCATA R: TACTTTTGTTGACCGCCTCC	(AG)_9_	168–176	—		MH017841
Phero_3893	F: TTCGGATCTACCATTCCGTC R: GTGCTTCAGCTGCATGTGTT	(CT)_9_	294–322	Unknown [*Picea sitchensis*]	2.90E‐126	MH017838
Phero_28905	F: TCTGTACACTGCACATGCCA R: GAGATCTTTCACCCACCCAA	(TA)_11_	207–249	—		MH017852
Phero_23143	F: CATCCAAAACAAGGCCTCTC R: TCTTAGGCGGTTGAGGAAAA	(TC)_12_	179–187	—		MH017851
Phero_11557	F: TCGAAATCGGCATGTGTTTA R: CACAAATCCCTTCTCCTCCA	(AT)_11_	221–259	Unknown [*Picea sitchensis*]	2.70E‐152	MH017843
Phero_4516	F: TCATGGCAGTCTTCTTCACG R: CCTCCCCTTTCTCCTGTCTC	(GAG)_8_	281–296	DUF1674 domain‐containing protein [*Acinetobacter baumannii*]	0.015	MH017839
Phero_12816	F: TGGCATTCATTTCTCTGCAT R: TACAAGTCAAACCATGGGCA	(GA)_9_	233–259	Serine/threonine protein phosphatase 2A 59 kDa regulatory subunit B′ gamma isoform‐like [*Manihot esculenta*]	0	MH017846
Phero_12324	F: TGTGGTCACAACACAGATCG R: GATCCGGAGTCCAATTCTGA	(GGA)_8_	298–304	PREDICTED: Transcription factor PAR1 [*Daucus carota* subsp. *sativus*]	8.00E‐08	MH017845
Phero_16341	F: GTCAGTCACGCCACAAGCTA R: TCTGCTACAACGCTTTCCCT	(AG)_12_	140–154	—		MH017848
Phero_15044	F: GTGTGCAGAGGGAGATGGAT R: ACCTTTTCTCCGCCAAAAAT	(AGG)_8_	132–140	Transcription factor PAR2‐like [*Asparagus officinalis*]	9.10E‐05	MH017847

Note

— = no BLASTX hits were found.

a All 15 loci were amplified using the same annealing temperature of 60°C.

**Table 2:** Genetic diversity of the 15 expressed sequence tag nuclear microsatellites in three populations of *Pherosphaera hookeriana* and nine samples of *P. fitzgeraldii*.^a^

Locus	*P. hookeriana*									*P. fitzgeraldii* (*n* = 9)		
	Gowan Brae (*n* = 30)			Wombat Moor (*n* = 30)			The Parthenon (*n* = 34)					
	*A*	*H*_o_	*H*_e_	*A*	*H*_o_	*H*_e_	*A*	*H*_o_	*H*_e_	*A*	*H*_o_	*H*_e_
Phero_20099	7	0.66	0.68	5	0.61	0.59	12	0.73	0.71	4	0.33	0.71
Phero_8380	10	0.73	0.83	10	0.75	0.76	13	0.85	0.85	1	0.00	0.00
Phero_11789	3	0.60	0.57	3	0.60	0.43	3	0.50	0.52	1	0.00	0.00
Phero_18747	2	0.43	0.38	4	0.23	0.21	5	0.56	0.53	1	0.00	0.00
Phero_6366	5	0.37	0.42	6	0.87	0.77	4	0.56	0.53	4	0.56	0.56
Phero_8339	4	0.63	0.67	5	0.63	0.64	3	0.71	0.62	1	0.00	0.00
Phero_3893	6	0.53	0.48	2	0.67	0.44	4	0.53	0.43	1	0.00	0.00
Phero_28905	6	0.77	0.81	8	0.80	0.79	7	0.68	0.76	1	0.00	0.00
Phero_23143	4	0.53	0.58	5	0.70	0.69	4	0.76	0.72	2	0.22	0.20
Phero_11557	10	0.77	0.84	11	0.80	0.81	13	0.91	0.84	2	0.11	0.10
Phero_4516	4	0.80	0.64	3	0.20	0.18	5	0.56	0.56	1	0.00	0.00
Phero_12816	5	0.76	0.74	5	0.73	0.74	9	0.68	0.70	2	0.11	0.10
Phero_12324	1	0.00	0.00	2	0.47	0.39	1	0.00	0.00	1	0.00	0.00
Phero_16341	2	0.03	0.25	5	0.69	0.63	3	0.26	0.37	1	0.00	0.00
Phero_15044	3	0.53	0.53	4	0.77	0.64	3	0.29	0.29	1	0.00	0.00
Average	4.80	0.54	0.56	5.20	0.63	0.58	5.93	0.57	0.56	1.60	0.09	0.11

Note

*A* = number of alleles; *H*_e_ = expected heterozygosity; *H*_o_ = observed heterozygosity; *n* = number of individuals sampled.

a Locality and voucher information are provided in Appendices 1 and 2.

## CONCLUSIONS

We developed 15 expressed sequence tag nuclear microsatellites for the Tasmanian vulnerable paleoendemic conifer *P. hookeriana*; these are the first such markers developed in the genus *Pherosphaera*. These loci will be useful for investigating the species' extant genetic diversity and structure, the impact of past environmental change, and the importance of asexual reproduction.

## DATA ACCESSIBILITY

Contigs from the de novo assembly are available from the Dryad Digital Repository (https://doi.org/10.5061/dryad.br73qg2; Worth et al., 2018).

## APPENDIX 1 Details of the three populations of *Pherosphaera hookeriana* used for assessing the genetic diversity of 15 nuclear expressed sequence tag microsatellites, including the location, the GPS coordinates, and accession numbers of existing preserved herbarium specimens representative of each population sampled in this study.

**Table:**

Species	Locality	*n*	GPS coordinates	Accession no.^a^^,^ b
*Pherosphaera hookeriana* W. Archer	Gowan Brae Road bridge over Nive River	30	42.05149°S, 146.44432°E	HO 115387
*P. hookeriana*	Wombat Moor, Mt. Field National Park	30	42.68621°S, 146.61094°E	CANB 885949.1
*P. hookeriana*	The Parthenon, Cradle Mountain–Lake St. Clair National Park	34	41.9571°S, 146.05014°E	HO 411672

Note

*n* = number of individuals sampled.

a Details of each specimen are available online at the Australasian Virtual Herbarium (http://avh.chah.org.au).

b CANB = Australian National Herbarium, Canberra, Australian Capital Territory, Australia; HO = Tasmanian Herbarium, Hobart, Tasmania, Australia (Thiers, 2018).

## APPENDIX 2 Details of the nine samples of *Pherosphaera fitzgeraldii* used for testing the transferability of the 15 nuclear EST microsatellites developed in *P. hookeriana*, including the location of the collection from which the sample was sourced, the natural population from which the sample was collected (if known), and the accession number of the living collection at Blue Mountains Botanic Garden.

**Table:**

Source	Natural population	Accession no.
Cultivated plant	Unknown	—
University of Tasmania collection	Unknown	—
Blue Mountains Botanic Garden	Wentworth Falls	13395
Blue Mountains Botanic Garden	Wentworth Falls	882396
Blue Mountains Botanic Garden	Leura Falls	913503
Blue Mountains Botanic Garden	Leura Falls	913502
Blue Mountains Botanic Garden	Leura Falls	913499
Blue Mountains Botanic Garden	Bonnie Doon Falls	AA800680
Blue Mountains Botanic Garden	Katoomba Falls	20000176

Note

— = accession number not available.
